# CHRIST: CD44-Incorporated Hepatocellular Carcinoma Risk Index Scoring Tool—A Novel Prognostic Scoring System for Hepatocellular Carcinoma Development and Aggressiveness

**DOI:** 10.3390/medicines9020014

**Published:** 2022-02-21

**Authors:** Ahmed Ali Khalifa, Nermeen Abdeen, Neveen L. Mikhael, Sawsan Elmalah, Ayman Elshayeb

**Affiliations:** 1 Department of Tropical Medicine, Clinical Pathology, Alexandria Faculty of Medicine, Alexandria 21433, Egypt; nermodean@gmail.com (N.A.); neveenlewis@hotmail.com (N.L.M.); drelmallah@gmail.com (S.E.); drayman65@yahoo.com (A.E.); 2 Department of Gastroenterology and Hepatology, Medical University of South Carolina, Charleston, SC 29425, USA

**Keywords:** CD44 rs187115, cluster of differentiation, cirrhosis, single-nucleotide polymorphism, hepatitis C virus

## Abstract

CD44 has been demonstrated to play a pivotal role in regulating tumor cell progression, including hepatocellular carcinoma (HCC) development. Here, we aimed to establish a scoring system to evaluate the risk of developing HCC utilizing CD44-rs187115 SNP polymorphism. A prospective cohort of 120 individuals was enrolled in four groups: 19 non-metastatic HCC patients, 21 metastatic, 40 patients with hepatitis C-related cirrhosis, and 40 controls. Allelic discrimination of the CD44-rs187115 gene polymorphism was assessed using TaqMan genotyping assay. HCC patients with CT/CC genotypes were more likely to have aggressive malignancy compared to TT carriers. A significant correlation was noted between the existence of CT/CC genotypes and tumor size, multicentricity, infiltration, portal vein thrombosis, and metastasis. A CD44-incorporated Hepatocellular Carcinoma Risk Index Scoring Tool (CHRIST) was formulated utilizing clinical and genetic variables. A score > 3 for HCC development demonstrated 87.5% sensitivity, 72.5% specificity, and a 76% positive predictive value (PPV) and 85% negative predictive value (NPV). Furthermore, a score > 5 for HCC metastasis demonstrated 90.4% sensitivity, 68.4% specificity, a 76% PPV and 86% NPV. A similarly significant score was noted following a six-month re-evaluation. We conclude that CD44-rs187115 may serve as a reliable prognostic biomarker for HCC and that the CHRIST prognostic model is highly predictive of the development of HCC and metastatic HCC.

## 1. Introduction

Despite the significant decline in hepatitis C virus (HCV) cases since the introduction of directly acting antivirals, the CDC estimates that nearly 2.4 million Americans were living with HCV infection in 2018 [1]. The incidence of HCC has been rising for the past three decades and is expected to keep rising until 2030 [2]. HCV is a leading cause of cirrhosis and hepatocellular carcinoma (HCC). A pivotal role of HCV in carcinogenesis is to establish a microenvironment that serves as a carcinogenic milieu [3]. Moreover, HCV proteins showed a direct carcinogenic effect [4]. The available data suggest that hepatic cancer stem cells (CSCs) are incorporated in the development of HCC. CSCs exhibit the capacity to induce and sustain tumor growth [5]. Liver-specific CSCs have been isolated in HCC by several cell-surface antigens including several clusters of differentiation (CD), such as CD44, CD133, and CD90. CD44 is the most correlated with HCC and was also correlated with aggressive behavior [6,7]. CD44-positive cells have been suggested to be involved in the epithelial-mesenchymal transition (EMT), a genetic process associated with cancer invasion and metastasis [8,9,10,11]. In addition, CD44+ cells engraft at high frequencies in mice and appear to possess enhanced chemoresistance [12,13]. The CD44 role in human cancer metastasis (and a possible therapeutic effect) was studied in several cancer cells, but CD44 gene single-nucleotide polymorphism (SNP) and its role in HCC development and clinical features remain poorly investigated [13,14,15,16,17]. Hence, we aimed to study a common SNP in the 3′untranslated region (UTR) of CD44 (identified as rs187115) and incorporate it into a scoring system to evaluate HCC behavior and aggressiveness.

## 2. Materials and Methods

### 2.1. Patient Selection

We prospectively identified all patients who presented to the Tropical Medicine department of Alexandria Medical University Hospital (AMUH) from January 2017 to August 2018. Patients with HCV-related cirrhosis with and without HCC were included if they were eligible. The exclusion criteria were concurrent hepatitis B virus infection, autoimmune diseases, diabetes mellitus, coronary heart disease, pulmonary fibrosis or active respiratory tract disease, hemodialysis, alcoholism, smoking history, and those with any malignancies other than HCC. Cirrhosis was diagnosed by imaging and liver biopsy and patients were subsequently staged using the Child-Pugh (CP) scoring system (a scoring classification of the severity of liver disease according to the degree of ascites, serum concentrations of bilirubin and albumin, prothrombin time, and degree of encephalopathy) and modified end-stage liver disease (MELD) score (a prospectively developed and validated cirrhosis-severity scoring system that uses a patient’s laboratory values for serum bilirubin, serum creatinine, and the international normalized ratio (INR) to predict their three-month survival) [18,19]. HCC was also diagnosed with either an imaging technique (triphasic computed tomography (CT) or magnetic resonance imaging (MRI)) or via liver biopsy and was staged according to the Barcelona Clinic Liver Cancer (BCLC) classification (comprising four stages based on the extent of the primary lesion, performance status, vascular invasion, and extrahepatic spread) [20].

To study the role of CD44 rs187115 gene polymorphisms among HCC patients, the distribution frequency of clinical features and the frequency of CD44 genotypes in HCC patients (compared to cirrhotic patients and the control group) were examined. The clinical features included patient demographics, laboratory variables, TNM staging, BCLC staging, tumor size, lymph node involvement, and distant metastasis.

The prospective study included 120 subjects who were classified as follows: group I, 21 cirrhotic patients with metastatic HCC; group II, 19 cirrhotic patients with non-metastatic HCC; group III, 40 patients with HCV-related liver cirrhosis; group IV, 40 healthy controls (matched for age, sex, socioeconomic conditions, and the exclusion criteria). The control group patients had no history of previous malignancy. The study was approved by the Alexandria Main University Hospital (AMUH) institutional review board, met all criteria for good clinical practice, and written informed consent was adequately obtained from all participants. All the patients’ demographics were collected via interviewer-administered questionnaires (initial visit (visit #1)), and the clinical data were collected on a separate visit (visit #2) or during their hospital course (if patients were admitted to the hospital).

### 2.2. DNA Extraction

Whole blood samples were collected from patients and controls into vacutainer tubes containing ethylenediaminetetraacetic acid (EDTA). Genomic deoxyribonucleic acid (DNA) was extracted using Purelink DNA mini blood kits (manufacturer: Thermofisher Scientific, company address: Middlesex County, MA, USA) according to the manufacturer’s instructions. The DNA quality was assessed on a nanodrop machine by measuring the OD at 260 and 280 nm. DNA samples were stored at −80 °C up to the time of genotyping.

### 2.3. Real-Time Polymerase Chain Reaction (PCR)

Allelic discrimination of the rs187115 polymorphism of the CD44 gene was assessed with Rotor-Gene Q (manufacturer: Qiagen, company address: Germantown, MD, USA) using a Taqman genotyping assay. The final volume for each reaction was 10 μL, containing 5 μL TaqMan Genotyping Master Mix, 1 μL TaqMan probe mix, and 10 ng genomic DNA completed with RNAase free water. The real-time PCR included an initial denaturation step at 95 °C for 10 min, followed by 40 cycles at 95 °C for 15 s, and then 1 min at 60 °C. Some of the samples were repeated inter-assay to validate the results from real-time PCR [21]. 

### 2.4. CHRIST: CD44-Incorporated HCC Risk Index Scoring Tool

We evaluated numerous clinical and laboratory variables collected for the patients, and several variables were found to be statistically significant in the bivariate analysis. These variables were cachexia, CP score, erythrocyte sedimentation rate (ESR), C-reactive protein (CRP), alpha-fetoprotein (AFP), and CD44 SNP. Those variables were then included in a multivariable logistic regression analysis to determine the variables correlating with high sensitivity and specificity in predicting HCC development and aggressiveness. Forward stepwise model selection was performed to obtain a parsimonious model of the predictors. ROC curves were generated using the proc logistic based on all available data, and the most appropriate cutoff for predicting HCC development and metastasis was obtained using tenfold cross-validation with test and training data. The results of the multivariate analysis were presented as odds ratios (ORs) along with the corresponding 95% confidence intervals (CIs). The model had a score of 10 points for predicting the probability of HCC development among cirrhotic patients and 8 points for predicting the probability of developing metastatic HCC among non-metastatic HCC patients. To assess the efficacy of the CHRIST score for predicting the probability of HCC development and predicting the probability of developing metastatic HCC among early-stage HCC cases, we followed up the patients for six months and re-evaluated the CHRIST score among the two groups of patients (40 cirrhotic patients with no HCC and 19 early-stage (non-metastatic) HCC). 

### 2.5. Statistical Analysis

The data were inputted on a computer and analyzed using IBM SPSS software package version 20.0. (IBM Corp., Armonk, NY, USA), and using SAS statistical package version 9.2 (SAS Institute, Cary, NC, USA). We described the qualitative data using numbers and percentages and the quantitative data using the range (minimum and maximum), mean, standard deviation, and median. The chi-squared test was used to compare different groups. Fisher’s Exact/Monte Carlo correction was used for the chi-squared test when more than 20% of the cells had an expected count below five. Student’s *t*-test for normally distributed quantitative variables was used to compare the two studied groups. We carried out the F-test or Mann–Whitney U test for normally and abnormally distributed quantitative variables, respectively. Kruskal–Wallis was used for abnormally distributed quantitative variables, to compare more than two studied groups. A receiver operating characteristic curve (ROC) was generated by plotting the sensitivity on the Y-axis versus 1-specificity on the X-axis at different cut-off values. The area under the curve demonstrates the diagnostic performance of the test. The odds ratio (OR) is used to calculate the ratio of the odds at the 95% confidence interval (CI) of an event happening in a risk group to the event occurring in a non-risk group. The adjusted odds ratios were estimated using multiple logistic regression models after controlling for age and gender in each comparison. Significance was judged at the 5% level.

## 3. Results

The mean age of the 120 subjects was 59 ± 4 years, and men represented the majority of the cohort (27 women, 22.5%). There was no statistical significance between the four groups regarding demographic variables (*p*-value of 0.650 for age and *p*-value of 0.857 for gender). Multiple clinical variables were evaluated including easily tired, dyspepsia, abdominal distension, jaundice, abdominal pain, weight loss, hematemesis/melena, hepatic encephalopathy. Patients with HCC (early and metastatic) showed statistically significant results compared to the cirrhosis group with regards: abdominal distension (*p* = 0.028), easy fatigue (*p* = 0.011), yellow discoloration (*p* = 0.012), dyspepsia (*p* = 0.037), abdominal pain (*p* = 0.045), and weight loss (*p* = 0.003). Among the HCC groups (1 and 2), the tumor size was significantly larger in the metastatic HCC group vs. non-metastatic (>5 cm, *p*-value = 0.001). Furthermore, malignant infiltration, portal vein thrombosis, and porta hepatis metastases were more likely in the metastatic HCC group compared to non-metastatic (Table 1). The CP score was C in the 20 cancer patients and 18 cirrhotic patients, while CP score B was seen in 20 cancer patients and 13 cirrhotic patients, and none of the patients with CP score A had a malignancy (*p* < 0.033). Nineteen patients of the non-metastatic HCC group were TNM I–IIIa while the remaining 16 patients of the malignancy group were metastatic and ranged from IIIa–IVa (*p* < 0.001) (Table 2). 

### 3.1. Comparison of the Four Studied Groups in Relation to CD44 Gene Polymorphism and Allele Frequency

In the control group, there were: 8 TT cases, 24 CT cases, and 8 CC cases. Among, this group, the CC allele carriers were found to match the TT and CT patients regarding demographic and clinical variables (including laboratory results). In the cirrhotic group, there were: 11 TT cases, 23 CT cases, and 6 CC cases. The non-metastatic HCC group had 10 TT cases and 9 CT cases, while the metastatic HCC group had 12 CT cases and 9 CC cases. There was a clinically significant difference for the CD44 gene polymorphism in different groups regarding the CD44 allele frequency (*p* < 0.001) (Table 3). 

### 3.2. Correlation between Different Genotypes Based on Tumor Behavior and Imaging Findings (CT/MRI)

**Figure 1:**
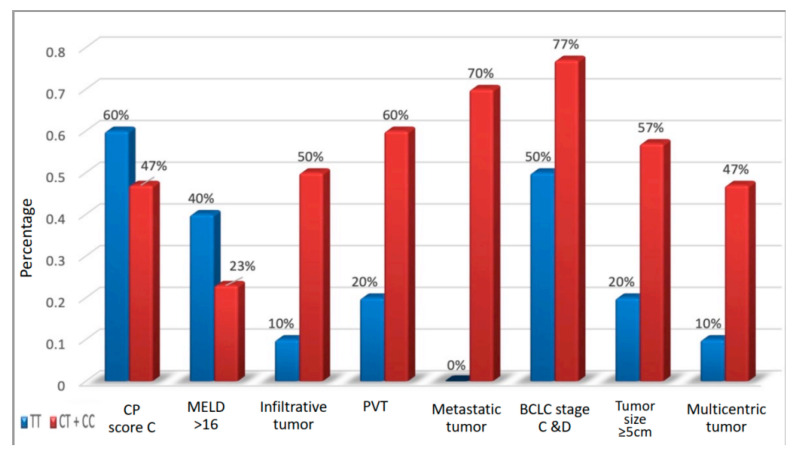
Correlation between different genotypes based on tumor behavior and computed tomography (CT) scan findings. Abbreviations: CP score: Child Pugh score; MELD: modified end-stage liver disease; PVT: portal vein thrombosis; BCLC staging system: Barcelona clinic liver cancer (BCLC) staging system.

CC/CT genotypes were the most common genotypes seen in the metastatic HCC group when compared to the non-metastatic HCC group, cirrhotic group, and control group (Figure 1). HCC patients with CT/CC genotypes were more prone to have an aggressive malignancy with a poorer prognosis compared to TT carriers. Hence, CT/CC genotypes were compared to TT carriers to identify the risk of an aggressive malignancy. A significant positive correlation was noted between the presence of CT/CC genotypes and tumor size (*p* = 0.044), multicentricity (*p* = 0.038), infiltrative behavior (*p* = 0.025), portal vein thrombosis (PVT) (*p* = 0.028), and porta hepatis lymph node metastases and distant metastases (*p* = 0.010). C allele expression was associated with a six-fold rise in the risk of PVT development (OR at 95% CI = 6.0 (1.08–33.27)) and eight-fold rise in the risk of metastases development (OR at 95% CI = 8.0 (1.4–44.9)), with *p* = 0.028 and 0.010, respectively (Table 4).

### 3.3. CHRIST Scoring System for Evaluation of HCC Development and Aggressiveness

We developed a score of 10 points for predicting the probability of HCC development among cirrhotic patients (as aforementioned in the methodology section). This score is composed of AFP > 200 ng/mL (four points), cachexia (one point), CP score > 8 (two points), CD44 (one point), ESR > 35 mm/h (one point), and CRP > 5 mg/L (one point). A score of more than three had a sensitivity of 87.5% and specificity of 72.5% at a positive predictive value (PPV) of 76% and negative predictive value (NPV) of 85%. After a six-month follow-up period, the CHRIST score was re-evaluated and a score of more than four was found to have the highest statistical value (sensitivity of 85% and specificity of 76% at PPV of 74% and NPV of 83%). In addition, we established a score of eight points for predicting the probability of developing metastatic HCC among early-stage HCC cases. This score is composed of AFP > 200 ng/mL (one point), cachexia (two points), portal vein thrombosis (one point), CD44 (two points), ESR > 35 mm/h (one point), and CRP > 5 mg/L (one point). A score of more than five had a sensitivity of 90.4% and specificity of 68.4% at a PPV of 76% and NPV of 86%. After a six-month follow-up period, the CHRIST score was re-evaluated for the early-stage HCC cohort (18 patients, as one patient died of septic shock secondary to pneumonia) and a score of more than five was found to have the highest statistical value (sensitivity of 89% and specificity of 70.5% at PPV of 78% and NPV of 80.3%).

## 4. Discussion

CD44 is a multi-structural, multifunctional, highly glycosylated transmembrane protein. CD44 levels were reported to be higher in numerous malignancies, chronic inflammatory diseases, and autoimmune disorders [22]. To understand the correlations between the clinicopathological features of HCC and the different CD44 rs187115 alleles, we analyzed the CD44 rs187115 gene polymorphism in 120 individuals. 

The main finding in our study was the significant correlation noted between CD44 rs187115 and the severity of HCC, where patients with at least one C allele at CD44 rs187115 were more likely to develop advanced HCC compared to wild-type (TT) carriers. Our results suggested that HCC patients with CC + CT alleles are more prone to progressing to metastatic HCC. Similarly, one study showed that CD44 might function as a prognostic marker for predicting HCC severity [23]. The study also reported that cirrhotic and HCC patients had high CD44 expression and reduced survival rates [23]. CD44’s significant correlation with HCC aggressiveness was also evaluated in another study that utilized formalin-fixed tissue sections from 107 resected liver cancers to detect the expression of various isoforms of CD44. The authors suggested that CD44 isoforms’ upregulation can be associated with poorly differentiated HCC and shortened survival [24]. In our HCC group, 66% of patients with CT + CC had LN metastases vs. only 22% of patients with the TT genotype (*p* = 0.010). In contrast, another study showed that 8.2% of HCC cases with CC + CT had LN metastases vs. 1.4% of HCC patients with the TT genotype (OR = 6.489, at 95% CI (0.703–59.899)) [25].

Interestingly, in the non-metastatic HCC group, tumor size was significantly smaller than that in the metastatic HCC group, with 21% of non-metastatic HCC patients having lesions bigger than 5 cm vs. 71% in the metastatic HCC group (*p* = 0.044). In contrast, one study reported that tumor size did not alter the CD44 expression pattern [26].

Another valuable observation was that 8 of the 40 controls (20%) had the CC allele. Despite that, these patients had no history of previous malignancies, and large genetic epidemiological studies in the field of cancer diseases have suggested the limited ability of polymorphic traits alone to refine the individual prognosis [27]. It would be interesting to evaluate the tumor behavior among these patients if they were to develop a malignant neoplasm. Furthermore, despite the distribution frequency of CC SNP among the three groups (HCC vs. cirrhosis vs. control group) being statistically significant (<0.001), it was found that the CC SNP frequency among the control group vs. the cirrhotic group (40 each) was insignificant (0.677). Yet, we suspect that identifying these patients early in the course of the disease will provide appropriate risk stratification and optimize treatment selection. For instance, the current data suggested that CD44 rs187115-positive adenocarcinoma patients who had surgical resection were less likely to have a good oncologic outcome after surgical treatment even at a resectable stage, and therefore, should be considered for alternative chemotherapy or radiotherapy protocols [28]. 

We were surprised to find that in the metastatic HCC group, 100% of the patients expressed CC + CT vs. 47% of patients in the non-metastatic HCC group (*p* < 0.001). On the contrary, one study reported no significant association with the tumor stage. Ref. [23] nevertheless, another study demonstrated a significant association between CD44 expression and the HCC stage (OR 2.38 at 95% CI (1.23–4.60); *p* = 0.01) [27]. This leads us to conclude that CC + CT was not only significantly correlated with the HCC stage but also with tumor multicentricity, PVT, infiltrative behavior, and lymphatic metastasis. In contrast, other studies showed that CC + CT was only significantly correlated with the tumor stage [25].

Using multivariate logistic regression analysis, six parameters were determined to be independent risk factors for the prediction of HCC development; these were CRP, ESR, AFP, cachexia, CP score, and CD44 rs187115 gene polymorphism. Moreover, we found that CRP, ESR, AFP, cachexia, and PVT were independent risk factors for the prediction of metastatic HCC development. These risk factors were re-evaluated after six months to determine their correlation with the disease evolution and further strengthen the predicting model’s value. The CHRIST scoring system suggested that cachexia may predict HCC and metastatic HCC development and progression. Similarly, research has suggested that the prognostic nutritional index (PNI) score may predict the prognosis in patients with malignancy regardless of the source [28,29]. In comparison, the cancer of the liver Italian program (CLIP) scoring system incorporates the CP score along with several tumor criteria (tumor morphology, AFP, and portal invasion). Nevertheless, the CLIP scoring system fails to utilize genomic biomarkers to assess the severity of liver tumors [30]. Our model is the first to integrate not only liver functions and patient-related variables but also tumor-related genetic elements that may be considered as an integral driving force in HCC development. The “five-gene” score (TAF9, RAMP3, HN1, KRT19, and RAN) was built to evaluate the genetic role in hepatic carcinoma and reported strong prognostic relevance [31]. 

We recognize several limitations to our study. The main limitation of our study is that no liver biopsies were done to assess CD44 expression at the hepatic tissue level. In comparison, HCC grading was done in one study and the authors concluded a significant positive correlation between CD44 expression and HCC grades [23]. Nevertheless, our study provides a novel scoring system that may utilize the clinical variables and blood samples to determine the risk of developing aggressive HCC, and hence, it may alter the current preventive and screening modalities. Secondly, the study population included patients in different phases of the disease evolution (cirrhosis, early-stage HCC, and metastatic HCC) and the values of AFP, ESR, and CRP may greatly differ at these different stages of the disease. However, we re-evaluated the CHRIST model after three months from the baseline in an attempt to reduce the variability of these laboratory variables over time. Thirdly, the study was restricted to a single center (all patients were Caucasians), which may limit the generalizability of its results. However, we highly encourage re-evaluating the genetic role of different populations for further score validation. Finally, the small sample size was a study limitation that might have limited the study power, and hence, we suggest adopting large sample sizes for future evaluations of the value of CD44 polymorphism as a tool to predict the development and metastasis of HCC.

In summary, we found that CD44 may play a pivotal role in identifying patients at a higher risk of developing HCC as well as those who are more likely to develop aggressive malignancy, and thus, CD44 may serve as a novel prognostic marker. The CHRIST scoring system proved to be useful for assessing the risk of early-stage HCC and metastatic HCC development. It is attractive to speculate that such a marker might be of high clinical significance for diagnosing high-risk HCC patients at an early stage, those who would greatly benefit from early intervention (e.g., liver transplantation), and hence may improve their management and outcomes.

## Figures and Tables

**Table 1:** Comparison between the four groups in relation to tumor characteristics.

	Hepatocellular Carcinoma		*p*-Value (For TT, CT, and CT)
	Non-Metastatic (*n* = 19)	Metastatic (*n* = 21)	
Infiltrating malignancy	1 (5.3%)	15 (71.4%)	<0.001
Portal vein thrombosis	0 (0%)	15 (71.4%)	<0.001
Porta hepatis metastasis	0 (0%)	16 (76.2%)	<0.001
BCLC			
0	0 (0%)	0 (0%)	0.001
A	2 (10.5%)	1 (4.8%)	
B	8 (42.1%)	1 (4.8%)	
C	0 (0%)	9 (42.9%)	
D	9 (47.4%)	10 (47.6%)	
Multicentric tumor	6 (31.6%)	9 (42.9%)	0.462
Tumor size			
<5	15 (78.9%)	6 (28.6%)	0.001
≥5	4 (21.1%)	15 (71.4%)	

Abbreviation—BCLC: Barcelona Clinic Liver Cancer.

**Table 2:** Liver disease scoring systems in patients with cirrhosis and liver malignancy.

	Hepatocellular Carcinoma		Cirrhotic (*n* = 40)	*p*-Value
	Non-Metastatic (*n* = 19)	Metastatic *(n* = 21)		
Child-Pugh score				
A	0 (0%)	0 (0%)	9 (22.5%)	0.033
B	9 (47.4%)	11 (52.4%)	13 (32.5%)	
C	10 (52.6%)	10 (47.6%)	18 (45%)	
MELD				
Mean ± SD	15.26 ± 3.45	13.52 ± 4.55	13.83 ± 5.15	0.170
TNM				
I	13 (%)	0 (0%)	-	<0.001
II	2 (%)	0 (0%)	-	
IIIa	4 (%)	0 (0%)	-	
IIIb & c	0 (0%)	11 (52.4%)	-	
IVa	0 (0%)	5 (23.8%)	-	
IVb	0 (0%)	5 (23.8%)	-	

Abbreviation—MELD: Modified end-stage liver disease; TNM staging: T describes the size of the tumor and any spread of cancer into nearby tissue, N describes spread of cancer to nearby lymph nodes, and M describes metastasis (spread of cancer to other parts of the body).

**Table 3:** Comparison of the four groups in relation to CD44 gene polymorphism and allele frequency.

	Hepatocellular Carcinoma		Cirrhotic (*n* = 40)	Control (*n* = 40)	*p*-Value
	Non-Metastatic (*n* = 19)	Metastatic (*n* = 21)			
CD44					
TT	10 (52.6%)	0 (0%)	11 (27.5%)	8 (20%)	<0.001
CT	9 (47.4%)	12 (57.1%)	23 (57.5%)	24 (60%)	
CC	0 (0%)	9 (42.9%)	6 (15%)	8 (20%)	
P_control_	0.013 *	0.033 *	0.677 *		
Significance between groups	*p*_1_ < 0.001 *, *p*_2_ = 0.077, *p*_3_ = 0.006 *				
Allele frequency					
T	29 (76.3%)	12 (28.6%)	45 (56.3%)	40 (50%)	<0.001
C	9 (23.7%)	30 (71.4%)	35 (43.8%)	40 (50%)	
P_control_	0.007 *	0.023 *	0.428 *		
Significance between groups	*p*_1_ < 0.001 **, *p*_2_ = 0.035 ***, *p*_3_ = 0.004 ****				

* P_control_: *p*-value for comparing control and other groups. ** *p*_1_: *p*-value for comparing non-metastatic HCC and metastatic HCC. *** *p*_2_: *p*-value for comparing non-metastatic HCC and cirrhotic. **** *p*_3_: *p*-value for comparing metastatic HCC and cirrhotic.

**Table 4:** Correlation between different genotypes and tumor behavior.

	CD44		*p*-Value	OR (95% CI)	Adjusted OR (95% CI)
	TT (*n* = 10)	CT + CC (*n* = 30)			
Child-Pugh score					
B 7,8,9	4 (40%)	16 (53.3%)	0.465	0.583 (0.136–2.49)	0.119 (0.01–1.313)
C ≥ 10	6(60%)	14 (46.7%)			
MELD					
<17	6 (60%)	23 (76.7%)	0.307	0.457 (0.10–2.091)	0.147 (0.02–1.92)
≥17	4 (40%)	7 (23.3%)			
BCLC					
0	0 (0%)	0 (0%)	0.160		
A	1 (10%)	2 (6.7%)			
B	4 (40%)	5 (16.7%)		-	-
C	0 (0%)	9 (30%)			
D	5 (50%)	14 (46.7%)			
A + B	5 (50%)	7 (23.3%)	0.111	3.28 (0.73–14.73)	2.5 (0.27–23.54)
C + D	5 (50%)	23 (76.7%)			
TNM					
I	8 (80%)	5 (16.7%)	0.002		
II	1 (10%)	1 (3.3%)			
IIIa	1 (10%)	3 (10%)		-	-
IIIb & c	0 (0%)	11 (36.7%)			
IVa	0 (0%)	5 (16.7%)			
IVb	0 (0%)	5 (16.7%)			
I, II, IIa	10 (100%)	9 (30%)	<0.001	-	-
IIIb & c, IVa, IVb	0 (0%)	21 (70%)			
Infiltrative tumor					
No	9 (90%)	15 (50%)	0.025	9.0 (1.011–80.13)	10.1 (1.07–104.3)
Yes	1 (10%)	15 (50%)			
Portal vein thrombosis					
No	8 (80%)	12 (40%)	0.028	6.0 (1.08–33.27)	4.58 (0.74–28.36)
Yes	2 (20%)	18 (60%)			
Metastasis					
No	10 (100%)	9 (30%)	0.010	8.0 (1.4–44.9)	6.71 (1.09–41.51)
Yes	0 (0%)	21 (70%)			
Tumor size					
<5	8 (80%)	13 (43.3%)	0.044	5.23 (1.04–28.91)	11.78 (1.12–123.3)
≥5	2 (20%)	17 (56.7%)			
Multicentricity					
No	9 (90%)	16 (53.3%)	0.038		

Abbreviation—MELD: modified end-stage liver disease; BCLC: Barcelona Clinic Liver Cancer; OR: odds ratio; CI; confidence interval.

## Abbreviations

AFP	Alpha-fetoprotein
AMUH	Alexandria Main University Hospital
BCLC	Barcelona Clinic Liver Cancer
CLIP	Cancer of the liver Italian program
CSCs	Cancer stem cells
CHRIST	CD44-incorporated hepatocellular carcinoma risk-index scoring tool
CP score	Child-Pugh score
CT	Computed tomography
CI	Confidence interval
CD	Cluster of differentiation
CRP	C-reactive protein
DNA	deoxyribonucleic acid
EMT	Epithelial-mesenchymal transition
ESR	Erythrocyte sedimentation rate
EDTA	Ethylenediaminetetraacetic acid
HCV	Hepatitis C virus
HCC	Hepatocellular carcinoma
MRI	Magnetic resonance imaging
MELD	Modified end-stage liver disease
NPV	Negative predictive value
OR	Odds ratio
PVT	Portal vein thrombosis
PCR	Polymerase chain reaction
PPV	Positive predictive value
PNI	Prognostic nutritional index
ROC curve	Receiver operating characteristic curve
UTR	Untranslated region
